# Error in Commentary

**DOI:** 10.1001/jamanetworkopen.2024.11449

**Published:** 2024-03-18

In the Invited Commentary “Possible Individual-, System-, and Policy-Level Contributors to Teen Pregnancy and Risk of Premature Mortality,”^1^ published March 14, 2024, there was an error describing the results of the study by Ray et al.^2^ In the first paragraph, the highest risk of premature death was among those whose pregnancies ended in livebirth, stillbirth, miscarriage, or ectopic pregnancy. This article has been corrected.^1^
